# Obstructive Sleep Apnea Is a Predictor of Abnormal Glucose Metabolism in Chronically Sleep Deprived Obese Adults

**DOI:** 10.1371/journal.pone.0065400

**Published:** 2013-05-29

**Authors:** Giovanni Cizza, Paolo Piaggi, Eliane A. Lucassen, Lilian de Jonge, Mary Walter, Megan S. Mattingly, Heather Kalish, Gyorgy Csako, Kristina I. Rother, the Sleep Extension Study Group

**Affiliations:** 1 Section on Neuroendocrinology of Obesity, National Institute of Diabetes, Digestive, and Kidney Disease, Bethesda, Maryland, United States of America; 2 Obesity Research Center, Endocrinology Unit, University Hospital of Pisa, Pisa, Italy; 3 Department of Molecular Cell Biology, Lab for Neurophysiology, Leiden University Medical Center, Leiden, The Netherlands; 4 Biomedical Engineering and Physical Science Shared Resource, National Institute of Biomedical and Bioengineering, Bethesda, Maryland, United States of America; 5 Department of Laboratory Medicine, Clinical Center, National Institutes of Health, Bethesda, Maryland, United States of America; 6 Section on Pediatric Diabetes and Metabolism, National Institute of Diabetes, Digestive, and Kidney Disease, Bethesda, Maryland, United States of America; Charité Universitätsmedizin Berlin, NeuroCure Clinical Research Center, Germany

## Abstract

**Context:**

Sleep abnormalities, including obstructive sleep apnea (OSA), have been associated with insulin resistance.

**Objective:**

To determine the relationship between sleep, including OSA, and glucose parameters in a prospectively assembled cohort of chronically sleep-deprived obese subjects.

**Design:**

Cross-sectional evaluation of a prospective cohort study.

**Setting:**

Tertiary Referral Research Clinical Center.

**Main Outcome Measure(s):**

Sleep duration and quality assessed by actigraphy, sleep diaries and questionnaires, OSA determined by a portable device; glucose metabolism assessed by oral glucose tolerance test (oGTT), and HbA_1_c concentrations in 96 obese individuals reporting sleeping less than 6.5 h on a regular basis.

**Results:**

Sixty % of subjects had an abnormal respiratory disturbance index (RDI≥5) and 44% of these subjects had abnormal oGTT results. Severity of OSA as assessed by RDI score was associated with fasting glucose (R = 0.325, *p* = 0.001) and fasting insulin levels (ρ = 0.217, *p* = 0.033). Subjects with moderate to severe OSA (RDI>15) had higher glucose concentrations at 120 min than those without OSA (RDI<5) (*p* = 0.017). Subjects with OSA also had significantly higher concentrations of plasma ACTH (*p* = 0.009). Several pro-inflammatory cytokines were higher in subjects with OSA (*p*<0.050). CRP levels were elevated in this sample, suggesting increased cardiovascular risk.

**Conclusions:**

OSA is associated with impaired glucose metabolism in obese, sleep deprived individuals. Since sleep apnea is common and frequently undiagnosed, health care providers should be aware of its occurrence and associated risks.

**Trial Registration:**

This study was conducted under the NIDDK protocol 06-DK-0036 and is listed in ClinicalTrials.gov NCT00261898

## Introduction

Several epidemiological studies have shown that people who report sleeping less than 6.5 h are at greater risk of gaining weight over time [1]. Furthermore, obesity and obstructive sleep apnea (OSA) frequently coexist: about 40% of obese individuals have OSA; conversely approximately 70% of individuals with OSA are obese [2], [3]. Similar to diabetes, OSA frequently goes undiagnosed [4]. Sleep duration and OSA may affect insulin resistance independently of body mass index (BMI) [5]. In addition, OSA is associated with decreased insulin sensitivity in lean, male subjects, suggesting that OSA per se may induce insulin resistance, independent of adiposity [6]. Several ongoing studies currently listed in Clinical Trials.gov and other similar web sites are addressing the relationship between OSA and glucose metabolism in obese subjects.

Seminal studies conducted by Van Cauter et al. demonstrated that acute sleep deprivation can induce insulin resistance in lean volunteers [7], [8]. Similarly, experimentally induced sleep fragmentation caused insulin resistance in healthy volunteers [9]. However, the effects of real life conditions, such as chronic sleep deprivation, on glucose metabolism have not been well characterized. The goal of the present report was to determine the relationship between short sleep, and OSA on glucose metabolism in a cohort of obese subjects reporting less than 6.5 h of nightly sleep.

## Methods

This analysis pertains to the Sleep Extension Study, a randomized, prospective, intervention trial of obese (BMI 30–55 kg/m^2^) men and premenopausal women 18 to 50 years old, who reported sleeping less than 6.5 h per night on average [10]. Subject recruitment took place between January 22, 2007, and June 28, 2011. All analyses presented here include data obtained at baseline prior to sleep intervention. Type 2 diabetes treated with insulin, a diagnosis of sleep disorders other than treated OSA, chronic use of sleep medications, chronic excess caffeine use, shift work and nocturnal occupations or current DSM-IV diagnoses, including anxiety, eating- or severe mood disorders, were exclusion criteria. Subjects taking oral hypoglycemic agents were allowed in the study.

### Ethics Statement

The study was conducted at the NIH Clinical Center in Bethesda, MD, USA after obtaining approval from the NIDDK Institutional Review Board (ClinicalTrials.gov identifier: NCT00261898). Each subject signed an approved written informed consent.

### Anthropometric Measurements

Height was measured to the nearest centimeter using a wall-mounted stadiometer (SECA 242, SECA North America East, Hanover, MD, USA) and weight was measured using a stand-on-scale in a hospital gown to the nearest 1/10th of a kg (SR555 SR Scales, SR Instruments, INC, Tonawanda, NY, USA). Waist circumference was measured at the midpoint between the inferior tip of the ribcage and the superior aspect of the iliac crest. Neck circumference was measured at the minimal circumference with the subjects’ head in the Frankfort Horizontal Plane.

### Body Composition Measurements

Dual-energy X-ray absorptiometry (DXA) for body composition assessment was performed with a Hologic DXA QDR 4500 (Hologic Inc., Bedford, MA, USA). Abdominal fat content and distribution was measured at the level of both L2–3 and L4–5, using a HiSpeed Advantage CT/I scanner (GE Medical Systems, Milwaukee, WI, USA) and analyzed on a SUN workstation using the MEDx image analysis software package (Sensor System, Sterling, VA, USA). Conventional (non-helical) 10 mm thick X-ray abdominal computed tomography images limited to the L2-3 and L4-5 levels were obtained at 120 kVp, with mAs adjusted according to patient size. Fully automatic processing of these images according to the method of Yao [11] resulted in measurements at these levels of visceral and subcutaneous adipose tissue areas, which were then summed and normalized by the imaged volume to estimate subcutaneous and visceral abdominal fat.

### Sleep Measures

Sleep was assessed by a combination of different methods. Subjects were instructed to wear a wrist activity monitor continuously for two weeks (Actiwatch-64, Mini Mitter/Respironics/Philips, Bend, OR, USA). Additional information on these instruments and data analysis was reported previously [12]. Sleep duration and sleep efficiency (percent of time asleep of total time spent in bed) were obtained from actigraphy. Self-reported sleep duration was derived from sleep diaries kept for two weeks, concurrent with the actigraphy measurements and from Question Four of the Pittsburgh Sleep Quality Index (PSQI), asking: “*During the past month, how many hours of actual sleep did you get at night?”.* The PSQI is a validated 21-item questionnaire that assesses subjective sleep quality over the past month [13]. PSQI scores range from 0 to 21, with higher scores indicating worse sleep quality. A score over 5 is considered the threshold for poor sleep quality. Each morning subjects were required to record the amount of sleep during the previous night in the sleep diaries. Daytime sleepiness was assessed by the Epworth Sleepiness Scale (ESS), a validated 8-item questionnaire [14]. ESS scores range from 0 to 24, with higher scores representing greater daytime sleepiness. A score greater than 10 indicates excessive daytime sleepiness.

The presence of sleep disordered breathing was evaluated over one night using a portable screening device (Apnea Risk Evaluation System, Advanced Brain Monitoring Inc., Carlsbad, CA, USA). This device provides an estimate of the respiratory disturbance index (RDI), which is the number of apneas and hypopneas per hour of sleep. An episode of apnea was defined as the complete cessation of airflow for at least 10 seconds. Hypopnea events were defined as at least 10 seconds with the airflow decreasing by more than 50% and with more than 3.5% oxygen desaturation, or more than 1% desaturation accompanied by at least one surrogate arousal indicator (head movement, changes in snoring, or changes in pulse rate) [15]. This device has demonstrated high sensitivity and specificity when validated against polysomnography [16].

### Glucose Metabolism Assessments

Fasting serum glucose and insulin were measured after a 10-h overnight fast. Each subject underwent a 75g oral glucose tolerance test (oGTT) during which plasma glucose and serum insulin levels were determined at 0, 30, 60, 90 and 120 min. Glucose levels ≥100 mg/dL at baseline and ≥140 mg/dL at 120 min of the OGTT were defined as abnormal, and the diagnosis of diabetes was made if glucose levels were ≥126 mg/dL at baseline and ≥200 mg/dL at 120 min. Insulin resistance was determined using the homeostasis model assessment for insulin resistance (HOMA): (fasting insulin (mU/L) * fasting plasma glucose (mg/dL))/405. The insulinogenic index was calculated with the following equation: (30 min insulin –0 min insulin)/(30 min glucose –0 min glucose). The AUC for glucose and insulin was calculated using the trapezoidal rule: 15 * (0 min plasma levels) +2 * (30 min, 60 min and 90 min plasma levels) +120 min plasma levels.

### Clinical Laboratory Analysis

Plasma glucose was determined with an enzymatic method. Plasma adrenocorticotropic hormone (ACTH), (total) serum cortisol, insulin, and growth hormone (GH) levels were measured with chemiluminescence immunoassays (Immulite 2000 and/or 2500 analyzers, Siemens). Urinary free cortisol (UFC) and catecholamines were collected in 24 h urine collection and measured using liquid chromatography-tandem mass spectrometry (LC-MS/MS) and high-performance liquid chromatography (HPLC), respectively.

Sixteen cytokines/chemokines were measured with an ELISA that uses the Quansys multiplex system (Quansys Biosciences, Logan, Utah, USA). All samples were run in duplicate. Values are reported in pg per mL after normalization to 1 µg total protein per mL of sample, to account for variations in the total protein content of the samples. CRP concentrations were measured in 87 subjects with a high sensitivity chemiluminescent immunometric assay with a detection limit of 0.1 mg/L (Immulite 2000, Siemens/DPC, Los Angeles, California, USA).

### Statistical Analysis

Descriptive statistics for each variable were calculated based on the presence of OSA according to a RDI cutoff value of 5 and on glucose status (i.e. normal and abnormal oGTT results). Statistical tests included Student’s *t* test and ANOVA for difference in means, Mann-Whitney U test for skewed variables, Fisher exact test and Pearson Chi-square test for difference in counts and frequency, respectively. The Kolmogorov-Smirnov test was used to assess normality of data; logarithmic transformations were applied for skewed variables before parametric statistical analyses (e.g. RDI and HOMA values). Pearson (r) and Spearman (ρ) correlation coefficients were used for Gaussian and skewed variables, respectively. The effect of OSA on the relationship between hormones and anthropometric parameters was assessed by analysis of covariance (ANCOVA). Multivariate regression models were also carried out. Data are presented as mean values ± standard deviation (SD) or median with interquartile range (IQR), as indicated. Analyses were performed using SAS (version 9.1.3, SAS Institute Inc., Cary, NC, USA), JMP (version 8.0, SAS Institute Inc., Cary, NC, USA) and SPSS (version 19. IBM SPSS North America. Chicago, IL, USA).

## Results

### Demographic and Anthropometric Characteristics According to Fasting Glucose and OGTT

Of the 125 randomized into the study, six subjects were excluded due to the use of oral hypoglycemic agents and 96 subjects had measurements of OSA available at the Randomization Visit. Demographic, anthropometric and life-style characteristics of these 96 subjects are shown in Table 1. Based on a RDI cut-off of 5, we divided subjects into two groups, with or without OSA. Approximately 40% of subjects had an RDI less than 5. Subjects with OSA were more often men, had higher body weight, waist and neck circumferences, similar subcutaneous fat but approximately twice as much visceral fat by CT.

**Table 1:** Demographic and anthropometric characteristics of the study subjects.

	No sleep apnea(RDI <5)(N = 38)	Sleep apnea(RDI ≥5)(N = 58)	p-value
Age (years)	39.9±6.6	42.5±5.9	**0.049**
Female	89.5%	69.5%	**0.041**
*Race*			0.646
Black	60.5%	50.8%	
White	34.2%	42.4%	
Other	5.3%	6.8%	
Years of education	16.5±2.4	15.8±2.5	0.162
Weight (kg)	99.7±16.6	110.4±19.8	**0.007**
Current or past smoking status			
Smoking history	13.1%	20.7%	0.496
Currently smoker	2.6%	10.3%	0.308
*Medications*			
Psychotropics (Prozac, Zoloft)	18.4%	5.2%	0.084
Hormonal contraceptives	18.4%	3.4%	**0.035**
Antihypertensives	10.5%	13.8%	0.871
Statins	5.3%	6.9%	0.909
Anti-asthma/allergy (Advair, Allegra)	5.3%	12.1%	0.448
Synthroid	2.6%	5.2%	0.919
BMI (kg/m^2^)	36.5±5.8	38.9±6.2	0.058
Body fat (%)	42.4±5.6	40.5±7.9	0.213
Body lean (%)	55.2±5.5	57.1±7.7	0.191
Waist circumference (cm)	108.7±12.3	116.4±12.4	**0.003**
Neck circumference (cm)	36.9±2.7	40.2±3.8	**<0.001**
Visceral fat by CT (cm^3^)	250.8±122.9	416.2±168.7	**<0.001**
Subcutaneous fat by CT (cm^3^)	921.6±294.8	888.3±296.1	0.606
Abdominal fat by CT (cm^3^)	1172.4±319.8	1304.5±317.7	0.060

Values in each cell are reported as mean ± SD or as percentage.

Subjects with OSA had also significantly higher fasting glucose, fasting insulin, HOMA index and HbA_1_C (Table 2). More precisely, 42% of the subjects with OSA had abnormal HOMA, 14% had abnormal fasting glucose, and 7.8% had abnormal HbA_1_c. Of the 58 subjects with OSA, 8 had a fasting glucose ≥126 mg/dL, 24 had glucose levels ≥140 mg/dL and 4 had glucose levels ≥200 mg/dL at 120 min of the OGTT test. None of the individuals without sleep apnea (SA) had either a fasting glucose ≥126 mg/dL or glucose levels ≥200 mg/dL at 120 min, however eight subjects had a 120 min glucose level ≥140 mg/dL.

**Table 2:** Glucowse characteristics for study subjects during fasting conditions and the oral glucose tolerance test.

	No sleep apnea(RDI <5)(N = 38)	Sleep apnea(RDI ≥5)(N = 58)	p-value
Fasting glucose (mg/dL)	84.9±6.7	91.3±11.5	**0.003**
% subjects with abnormal fasting glucose (≥100 mg/dL)	2.6%	13.6%	0.144
Fasting insulin (mU/L)	8.8±6.4	11.7±6.7	**0.037**
% subjects with abnormal oGTT	21.1%	44.1%	**0.036**
HOMA index^1^	1.4 (0.8–2.4)	2.4 (1.5–3.8)	**0.003**
% subjects with abnormal HOMA (≥2.5)	23.7%	42.4%	0.097
HbA_1_c (%)	5.5±0.5	5.8±0.6	**0.019**
% Subjects with abnormal HbA_1_c (>6.4%)	2.6%	6.8%	0.661

Unless otherwise stated, values in each cell are reported as mean ± SD or as percentage.

1 Values are reported as median with interquartile range due to skewed distribution.

**Figure 1:**
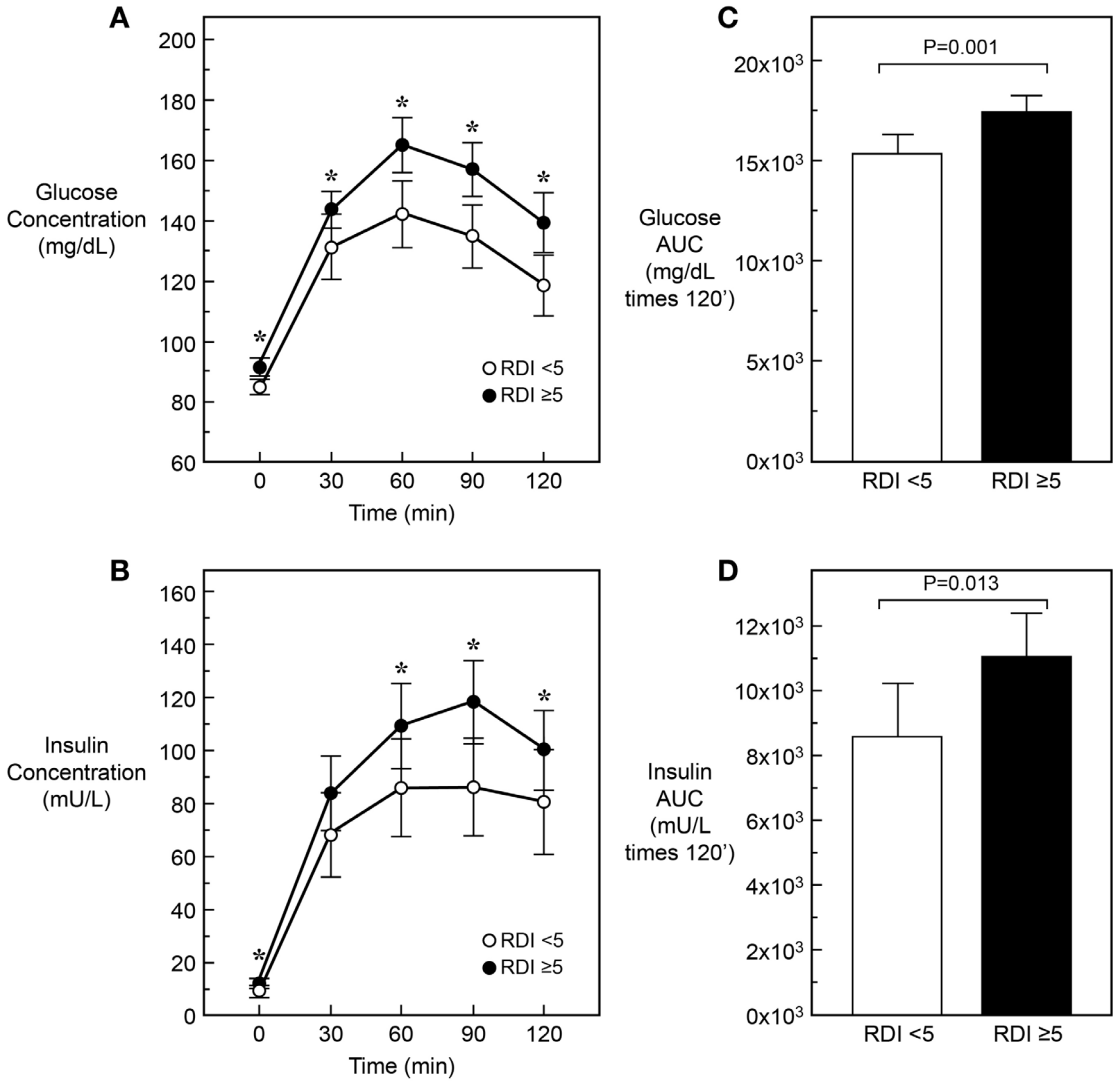
Oral glucose tolerance test. Glucose (Panel A) and insulin (Panel B) concentrations during 120-min OGTT in patients with a sleep apnea diagnosis (RDI ≥5, white circles, N = 58) and without a sleep apnea diagnosis (RDI<5, black circles, N = 38). The time-integrated area under the curve (AUC) for glucose and insulin are shown in panel C and D, respectively.

Fig. 1 depicts glucose (upper panels A–C) and insulin (lower panels B-D) concentrations and AUC during the oGTT in subjects with and without OSA. Subjects with OSA had significantly higher plasma glucose levels at each time point, and higher insulin at baseline, 60, 90 and 120 min; they had also approximately 10% higher glucose and insulin AUCs.

### Sleep Characteristics

Because of the inclusion criteria, subjects in both groups had short sleep duration, approximately 6.5h by sleep diary and 6h by actigraphy (Table 3). Average sleep efficiency was poor, 80%, approximately 80% of subjects had low sleep quality as indicated by a score greater than 5 on the PSQI scale, and 25% experienced sleepiness during the day, as indicated by a score greater than 10 on the ESS scale. Subjects with OSA had a median of 13 episodes of OSA per hour and slightly lower oxygen saturation than subjects without OSA. There were no group differences in sleep efficiency and sleepiness scores.

**Table 3:** Sleep characteristics of study subjects.

	No sleep apnea(RDI <5)(N = 38)	Sleep apnea(RDI ≥5)(N = 58)	p-value
Self-reported sleep duration (min/night)	385±54	387±42	0.868
Actigraphy sleep duration (min/night)	365±48	350±49	0.171
Actigraphy sleep efficiency (%)	80.8±4.9	80.0±6.8	0.559
PSQI global score	7.8±2.3	7.9±2.7	0.836
PSQI abnormal (>5) score	81.1%	84.5%	0.876
ESS score	8.7±4.7	8.1±4.4	0.504
ESS abnormal (>10) score	34.3%	26.3%	0.541
RDI (events/h)^1^	2 (1–4)	13 (8–20)	**<0.001**
Normal (RDI <5)	100%	0%	**<0.001**
Mild sleep apnea (RDI: 5–15)	0%	66.1%	**<0.001**
Moderate sleep apnea (RDI: 16–30)	0%	22.0%	**<0.001**
Severe sleep apnea (RDI >30)	0%	11.9%	**<0.001**
Saturation of peripheral oxygen (%)	96.8±0.9	95.6±2.3	**0.002**

Unless otherwise stated, values in each cell are reported as mean ± SD or as percentage.

1 RDI values are reported as median with interquartile range due to its skewed distribution.

**Figure 2:**
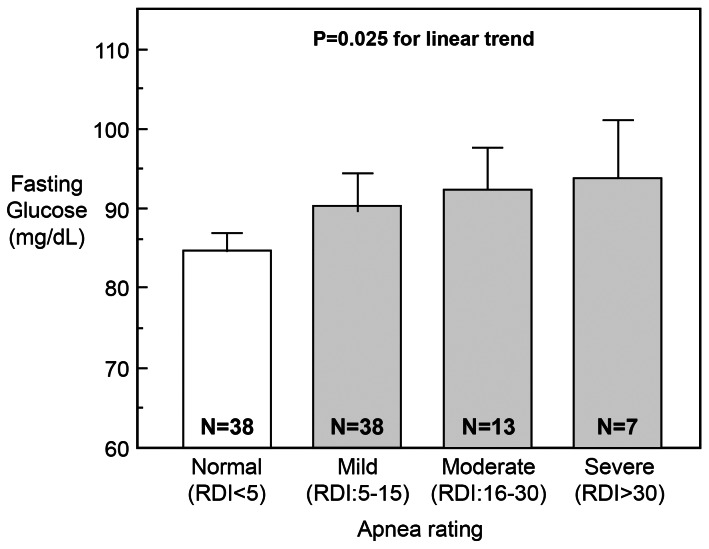
Fasting glucose concentration increases with sleep apnea severity as quantified by the RDI score. Fasting glucose concentrations rose progressively from subjects without sleep apnea (84.9±6.7 mg/dL; mean±SD) to those with mild (90.4±12.9 mg/dL), moderate (92.5±8.9 mg/dL), and severe (93.9±17.9 mg/dL) sleep apnea (test for trend: *p* = 0.025). Data are presented as mean with 95% CI.

Fig. 2 depicts average fasting glucose concentrations with increasing classes of severity of OSA. Fasting glucose concentrations rose progressively from subjects without OSA to those with mild, moderate and severe OSA. Subjects with moderate to severe OSA (RDI>15) had higher glucose levels at 120 min than subjects without OSA (*p* = 0.017).

**Figure 3:**
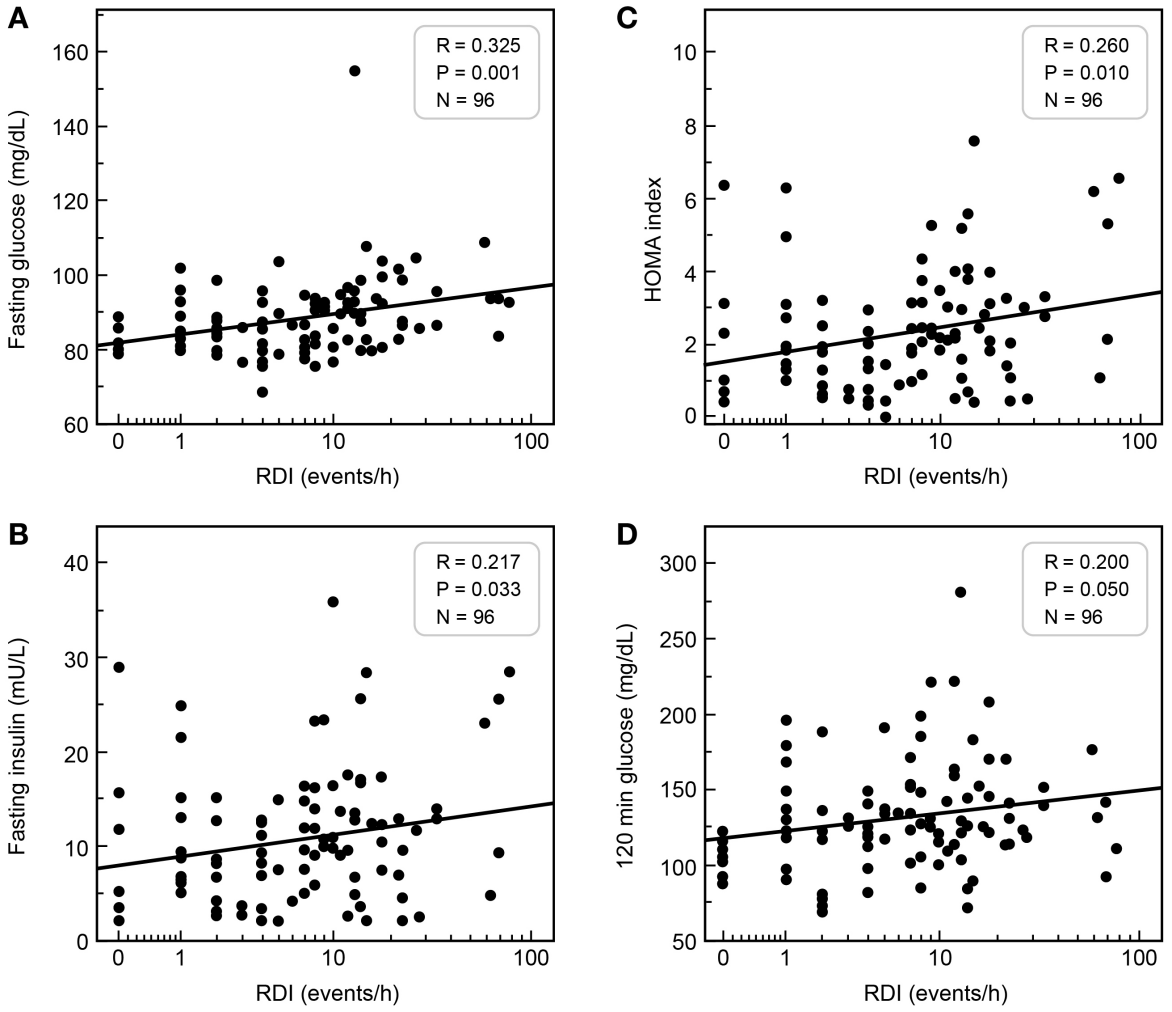
Relationship between RDI and fasting glucose (Panel A), fasting insulin (Panel B), HOMA index (Panel C), and 120-min glucose (Panel D). RDI is reported on a *safe*-logarithmic scale, namely, LOG_10_(1+ RDI).

Fig. 3 illustrates the relationship of log RDI and fasting glucose and insulin concentrations, HOMA, and 120 min glucose: log RDI was directly related in a significant fashion to each one of these parameters.

### Inflammatory/immune Characteristics

Subjects with OSA had higher levels of IL-2, IL-4, IL-5, IL-6, IL-8, IL-13, and IFN-gamma and tended to have higher levels of IL-15 and TNF-beta (Table 4). No significant differences between the two groups were observed in the remaining cytokines, as well as in CRP concentrations. CRP concentrations for the whole sample were: 4.78 mg/L (1.75–8.65) median and interquartile range.

**Table 4:** Inflammatory/immune characteristics of study subjects.

	No sleep apnea(RDI <5)(N = 38)	Sleep apnea(RDI ≥5)(N = 58)	p-value
IL-1a (pg/mL)	14.1 (9.5–18.9)	13.6 (0.3–20.7)	0.803
IL-1b (pg/mL)	30.1 (22.5–39.7)	32.4 (24.6–47.3)	0.418
IL-2 (pg/mL)	7.4 (4.5–12.3)	10.1 (7.5–15.7)	**0.047**
IL-4 (pg/mL)	3.2 (2.3–4.6)	4.1 (3.1–6.4)	**0.038**
IL-5 (pg/mL)	6.9 (4.2–9.0)	8.2 (5.3–10.7)	**0.033**
IL-6 (pg/mL)	4.5 (2.6–9.0)	5.8 (4.4–9.3)	**0.041**
IL-8 (pg/mL)	10.8 (4.7–15.4)	12.2 (7.7–21.8)	**0.050**
IL-10 (pg/mL)	7.8 (4.6–12.0)	8.0 (5.8–11.3)	0.615
IL-12 (pg/mL)	10.6 (5.0–12.8)	11.5 (7.8–14.1)	0.391
IL-13 (pg/mL)	13.2 (10.2–15.0)	14.3 (11.8–17.5)	**0.039**
IL-15 (pg/mL)	12.4 (9.9–14.7)	13.3 (11.3–15.5)	0.102
IL-17 (pg/mL)	13.9 (10.6–17.8)	14.3 (12.2–17.5)	0.358
IL-23 (pg/mL)	134.7 (95.2–204.2)	145.7 (123.9–187.2)	0.268
IFN-gamma (pg/mL)	27.0 (23.4–33.1)	34.0 (27.6–40.4)	**<0.001**
TNF-alfa (pg/mL)	16.6 (13.1–22.0)	19.9 (14.5–24.8)	0.135
TNF-beta (pg/mL)	14.1 (11.2–16.7)	15.5 (12.8–18.6)	0.095
C-Reactive Protein* (mg/L)*Logarithimic values (mean±SD)*	3.81 (1.09–8.65)0.53±0.54	5.27 (2.06–8.46)0.61±0.45	0.5840.448
<3.00 mg/L	1.20 (0.70–1.75)	1.77 (0.76–2.27)	0.339
3.00–9.99 mg/L	6.01 (4.59–7.49)	6.40 (5.30–8.44)	0.582
≥10.00 mg/L	16.40 (13.45−22.35)	14.90 (11.40−16.10)	0.114

Values are reported as median with interquartile range due to skewed distribution.

* N = 87

### Hormonal Characteristics

Subjects with OSA had approximately 36% higher plasma ACTH levels in the setting of similar serum cortisol and UFC (Table 5). In addition, they had 16% higher 24 h urinary norepinephrine levels. GH concentrations were significantly lower in subjects with OSA while no differences were observed in IGF-1 plasma concentrations.

**Table 5:** Hormonal characteristics of study subjects.

	No sleepapnea(RDI <5)(N = 38)	Sleep apnea(RDI ≥5)(N = 58)	p-value
Plasma morning ACTH (pg/mL)^1^	15.2 (11.7−21.5)	20.0 (14.5−27.4)	**0.009**
Serum morning cortisol (µg/dL)	9.1±4.3	10.0±4.6	0.361
UFC (µg/24 h)	18.0±10.6	20.7±14.6	0.322
Urinary norepinephrine (µg/24 h)	39.1±18.0	45.6±17.9	0.093
Urinary epinephrine (µg/24 h)	4.4±2.9	4.2±3.0	0.807
Urinary dopamine (µg/24 h)	249.0±87.8	259.3±98.4	0.614
Serum GH (ng/mL)^1^	0.70 (0.20−2.30)	0.20 (0.10−0.45)	**0.002**
IGF-1 (ng/mL)	141.2±52.6	135.0±43.6	0.530

1 Values are reported as median with interquartile range due to skewed distribution.

a To convert gravimetric units for hormones to SI units, use the following conversion factors: cortisol, µg/dl * 27.59 =  nmol/l; epinephrine, µg/24 h * 5.46 =  nmol/24 h; NE, µg/24 h * 5.91 =  nmol/24 h; corticotropin (ACTH), pg/ml * 0.22 =  pmol/l; and dopamine, µg/24 h *6.58 =  nmol/24 h; IGF-1, ng/mL*0.131 =  nmol/L.

### Multivariate Analyses of Determinants of Fasting Glucose

In a simple regression model, approximately 11% of the variability in fasting glucose was explained by RDI (Table 6). Incremental adjustments for visceral fat (Step 1), visceral fat and age (Step 2), as well as visceral fat, age, gender and sleep duration (Step 3) in multivariate models, increased the variability accounted for by the model from 11% to 16%. Based on this regression analysis, 10 episodes of OSA would increase fasting glucose by 2.01 mg/dL and a 10% increase in visceral fat would increase fasting glucose by 0.86 mg/dL. Similar results were obtained when BMI was used instead of visceral fat (data not shown).

**Table 6:** Multivariate statistical models of sleep apnea (as quantified by RDI score) predicting fasting glucose concentration.

Dependent variable:Fasting Glucose (mg/dL)	Step 0unadjusted	Step 1adjusted for visceral fat	Step 2adjusted for visceralfat and age	Step 3adjusted for visceral fat, age, gender and sleep duration
*Intercept (mg/dL)*	82.4(78.1 to 86.7)	80.2(74.9 to 85.4)	78.4(64.1 to 92.6)	77.1(52.8 to 101.3)
*Goodness of fit*	**R^2^ = 0.106, ** ***p*** ** = 0.001**	**R^2^ = 0.144, ** ***p*** ** = 0.001**	**R^2^ = 0.144, ** ***p*** ** = 0.005**	**R^2^ = 0.162, ** ***p*** ** = 0.014**
RDI(logarithmic values)	**7.3** * **(3.0 to 11.6)**	**5.4** * **(0.3 to 10.5)**	**5.3** * **(0.2 to 10.5)**	**5.8** * **(0.3 to 11.3)**
Visceral fat by CT (cm^3^)		0.012(−0.002 to 0.026)	0.012(−0.002 to 0.026)	0.016(−0.001 to 0.032)
Age(yrs)			0.047(−0.301 to 0.395)	0.084(−0.277 to 0.446)
Gender(Female = 0, Male = 1)				−2.7(−8.7 to 3.2)
Actigraphy sleep duration (min/night)				0.004(−0.044 to 0.052)

Beta coefficients in each cell were calculated after adjustment for the other independent variables in the multivariate model, and reported as mean values with 95% CI.

* =  *p*<0.05.

## Discussion

In this cohort of middle aged, obese men and premenopausal women who reported sleeping less than 6.5h on a regular basis, approximately 60% had OSA and 40% had abnormal glucose metabolisms. Prior to enrollment, the majority of subjects were unaware of having SA. SA is associated with worse glycemia and insulin resistance, known predictors of diabetes and cardiovascular disease.

The mechanisms of the relationship between abnormal glucose metabolism and OSA are incompletely understood. OSA is associated with chronic intermittent hypoxia and sleep loss due to sleep fragmentation, both of which contribute to insulin resistance [9], [17]. Inducement of a pro-inflammatory state, generation of reactive oxygen species, increased sympathetic tone, and alterations in the hypothalamus-pituitary-adrenal axis, are all putative mechanisms linking OSA to changes in glucose metabolism [18], [19]. In addition, beta cells are highly sensitive to hypoxia, and the subsequent shift to glycolytic metabolism favors insulin resistance. In addition, exposure to altitude worsens insulin sensitivity, as indicated by a lower glucose AUC in healthy subjects [20].

Consistent with the above-proposed mechanisms, we found, using a multiplex assay with high sensitivity, that a wide range of functionally related cytokines and chemokines were elevated in patients with OSA. Specifically, subjects with OSA had higher circulating concentrations of IL-2, IL-4, IL-5, IL-6, IL-8, IFN-gamma, and TNF-alpha and tended to have higher concentrations of TNF-beta, clearly indicating an activation of the Th-1 and Th-2 immune response. Increased cytokine levels have previously been observed in association with sleep deprivation, OSA, obesity, and insulin resistance in smaller samples using a limited number of cytokines [21]. The anti-inflammatory cytokine IL-10 was not elevated in subjects with OSA, indicating the lack of a counter-regulatory response. Our findings add novel information on IL-2, IL-4, IL-5, IL-10, IL-13, and IL-15. Since cytokines are secreted in a cascade, determination of one or a few cytokines may provide incomplete and potentially misleading information. In addition, traditional ELISA assays may not be sensitive enough to reliably determine cytokine concentrations in subjects without an overt clinical inflammatory or autoimmune condition. C-reactive protein (CRP) concentrations were abnormal in 66% of subjects. The synthesis of the acute phase mediator CRP by the liver is triggered by IL-6 and other cytokines. CRP is an accepted marker of cardiovascular risk with values ≥3-mg/L indicating increased risk [22].

Subjects with OSA had 60% more visceral fat by CT. Visceral fat is a major source of inflammatory cytokines. Inflammatory cytokines are produced by adipocytes, as well as by a heterogeneous aggregate of immune cells made up mostly of macrophages, but also by T and NK cells embedded among adipocytes. Hypoxia represents a potent stimulus for cytokine production from the adipose tissue *via* the intracellular pathways of c-Jun and ATF2 [23]. Of note, mean oxygen saturation was within normal limits in subjects with OSA and only 1% lower than in subjects without OSA. These findings suggest that even a small decrease in oxygen desaturation within the normal range of may have a significant impact in obese, sleep-deprived subjects. Based on our multivariate model, the effect size of the number of episodes of OSA on fasting glucose was much more important than that of visceral fat. Our observation has clinical implications for the prevention and treatment of diabetes: in obese, chronically sleep deprived subjects, treating OSA may be more effective than losing abdominal fat.

There were some hormonal differences between subjects with and without OSA. Morning plasma ACTH and urinary norepinephrine were higher in subjects with OSA, suggesting an activation of the stress system. Single time-point serum GH levels were significantly lower in subjects with SA, which is compatible with the decreased activity of the GH axis in obese subjects [24], but IGF-1 levels were normal in both groups. These findings should be considered exploratory, given that both the HPA and the GH axes were studied under basal conditions and hormonal measurements conducted on single time-point samples.

At variance with previous reports, sleep duration and glucose parameters were not related in our sample [2], [3], [5], [25], [26]. Most of the existing studies applied protocols of acute sleep deprivation to healthy, non- chronically sleep-deprived lean subjects [27]. We included only obese and chronically sleep deprived subjects and as such it is likely that chronic sleep deprivation may have already produced its negative effects on glucose metabolism.

Several studies have reported on the relationship between OSA and glucose metabolism [2], [3], [5], [25], [26], [28], [29]. The prevalence of OSA in our cohort was higher than the prevalence reported in the Wisconsin Sleep Cohort Study [4]. In the latter study, representing a large, random sample of 30 to 60 year old state agency employees reporting habitual snoring, 9% of women and 24% of men had OSA. Our higher prevalence may be due to the fact that our sample consisted exclusively of obese subjects with short sleep duration. In addition, 60% of our subjects were African American, an ethnic group in which both obesity and OSA are common.

Polysomnography is the gold standard for diagnosing OSA. This method requires an overnight stay in the hospital, is associated with considerable cost and inconvenience and may interfere with sleep. Therefore, in the current study we used for practical purposes portable devices designed for home use. Given the already high and increasing prevalence of OSA, we recommend using these devices more routinely to make large-scale screening practical, while polysomnography should be reserved for more complicated cases in which a formal sleep study may be required. By analogy, while for research purposes insulin resistance is determined by insulin clamp, for clinical purposes determination of fasting glucose and insulin are used. As recently stated by the Centers for Disease Control and Prevention (CDC), universal screening for type 2 diabetes in middle aged African-American, a segment of the population very similar to our sample, is considered very cost-effective [30]; similarly, diagnosis and treatment of OSA is considered cost-effective [31]. It is therefore likely that screening would be quite advantageous in subjects with OSA and obesity. An effort should be made to further develop simple devices that could be used at home; in addition, algorithms using parameters such as snoring, the degree of severity of sleepiness, neck circumference and other measures, should be used for the screening and diagnosis of OSA.

Study limitations include the fact that we did not characterize sleep architecture, that this cross-sectional evaluation was not designed to assess causality, and that the composition of the sample did not allow analyses of gender or ethnic differences. In addition, measurements of OSA were available in 96 of 125 subjects randomized, whereas CRP measurements were available in 87 subjects. As study merits, we would like to note the relatively large and well characterized sample in a real life setting, and the determination of a large variety of cytokines measured simultaneously with a sensitive assay. Finally, this cohort was exclusively composed of obese subjects.

In summary, OSA was more prevalent in our chronically sleep-deprived obese population than previously reported, its presence and severity were closely linked to abnormalities of glucose metabolism and insulin resistance to a greater extent than abdominal fat. OSA should be suspected, diagnosed and treated early in this population. The general public and healthcare providers should be made more aware of the health consequences of OSA, including abnormal glucose metabolism. Ongoing and future studies determining optimal interventions for OSA to improve glycemia and lower cardiometabolic risk are of upmost importance.
